# Moving as a group imposes constraints on the energetic efficiency of movement

**DOI:** 10.1098/rspb.2024.2760

**Published:** 2025-02-19

**Authors:** James A. Klarevas-Irby, Brendah Nyaguthii, Damien R. Farine

**Affiliations:** ^1^ Department of Migration, Max Planck Institute of Animal Behavior, Radolfzell, Germany; ^2^ Department of Evolutionary Biology and Environmental Studies, University of Zurich, Zurich, Switzerland; ^3^ Division of Ecology and Evolution, Research School of Biology, Australian National University, Canberra, Australia; ^4^ Mpala Research Centre, Nanyuki, Kenya; ^5^ Department of Ornithology, National Museums of Kenya, Nairobi, Kenya; ^6^ Department of Collective Behavior, Max Planck Institute of Animal Behavior, Konstanz, Germany

**Keywords:** animal behaviour, collective movement, energetic efficiency, GPS tracking, movement ecology, vulturine guineafowl

## Abstract

Movement is a key part of life for many species. In solitary animals, the energetic costs of movement can be mitigated through energetically efficient strategies that produce faster, straighter movements. However, little is known about whether moving as part of a collective enhances or limits the ability of individual group members to express such strategies. Drawing on 6 years of population-level, high-resolution (1 Hz) GPS tracking of group-living vulturine guineafowl (*Acryllium vulturinum*), we detected 886 events from 94 tagged individuals where their groups made large, range-shifting displacements in response to changing environmental conditions. We contrasted these movements with data from 94 similarly large displacement events by 19 lone, dispersing individuals. Our results suggest that individuals in groups can significantly reduce their energetic cost of transport when making large displacements (15.3% more efficient relative to their normal daily ranging) by increasing the speed and straightness of their movements. However, even during their most efficient movements, individuals in groups could not achieve or maintain comparable increases in speed to lone individuals, resulting in significantly limited efficiency gains (35.7% less efficient than solitary individuals). Overall, this study provides evidence for a substantial energetic cost arising from collective movement.

## 1. Introduction

Individuals in groups have to solve the same challenges as solitary individuals, from finding resources and mates [1,2] to escaping from predators [3,4]. While group living can benefit individuals in terms of detecting predators [5], improved navigation [6,7] and collective memory [8], group members also have to overcome the challenges of coordinating their actions to remain cohesive. Disagreements among group members in terms of preferred movement directions [9] or movement capabilities [10] can require some individuals to compromise their own optimal strategy in order to remain with the group [11]. Such compromises are likely to impose costs, but to date, the nature and extent of these costs remain largely overlooked. One potential cost that individuals in collectives could pay is through an increased energetic cost of transport—a measure that captures how much energy they use to move a given distance. In many species, individuals can mitigate some of the cost incurred when moving large distances by using strategies that make movements more efficient (i.e. by reducing the cost of transport). For example, migrating [12–14] and dispersing [15–19] animals can conserve energy over large distances by increasing the speed and straightness of their movements relative to their normal daily movements [20]. A key question is whether groups can achieve similar increases in efficiency to lone individuals when making movements over large distances or whether the challenges of coordinating actions as a group limit the ability of individual group members to express energetically efficient movement strategies.

Allometric models of the energetic costs of terrestrial locomotion [21,22] reveal that greater movement speeds are typically more efficient (have a lower cost of transport) because the increased instantaneous energetic investments (how much more oxygen the animal consumes per second) are smaller than the increase in speed (how much more distance it covers per second). Thus, terrestrial animals use disproportionately less energy per unit of distance travelled the faster they move (with the exception of very small species [22]). The decrease in the cost of transport with movement speed can help explain some patterns in terms of how groups move, such as in heterogeneous groups where differences in body size correspond to differences in movement capability.

In olive baboons (*Papio anubis*), smaller individuals have a slower natural gait than larger individuals, but group speed is determined by the larger individuals, with smaller group members having to speed up [10]. This pattern makes sense in the context of the cost of transport because smaller individuals increase their efficiency by speeding up (spending relatively less energy to cover the same distance), whereas larger individuals would incur a higher cost of transport if they slowed down to the speed of the slowest individuals. Thus, compromise in animal groups may entail an increase in the instantaneous costs paid by some individuals, but ultimately increase the overall efficiency of group members’ movements.

Other forms of compromise, such as making collective decisions about where to go next (e.g. through shared decision-making [9]) could have a greater impact on movement costs. For example, in many terrestrial animal groups, individuals attempt to influence the direction of group movements by initiating (moving in a desired direction) [23]. Failing in an initiation (which is common) would then require a net output of energy with no net displacement achieved, thereby reducing movement efficiency. Further, resolving conflicts among group members about where to go (e.g. when multiple individuals initiate in different directions) may cause some—or all—group members to slow down or stop entirely. This continuous process of decision-making ‘on the go’ [24] would then reduce the speed and the continuity of movement in terrestrial animal groups. As a result, we expect that moving as part of a group could be less efficient for individuals compared with moving alone, potentially resulting in a greater cost of transport.

While we predict that moving in groups should impose some constraints on efficiency, it is important to consider that the net costs of displacement are determined not only by the speed of movement but also by the straightness of the path taken between two points. All else being equal, a more tortuous path (such as when making exploratory movements [25]) involves covering more ground and thus spending more total time moving than taking a straighter path between two points—inducing a net energy cost. Here, moving in a group could be beneficial. For example, pooling directional uncertainty across multiple individuals can yield greater navigational accuracy (the ‘many wrongs’ hypothesis [7,26]). Studies of pigeons (*Columba livia*) [6,27,28] and humans [29] navigating towards a target have confirmed that individuals can have a more direct path when moving in a group than when moving alone. Such a relationship between the distance travelled and group size is likely to be also true in a broader range of systems and contexts, such as navigation during nomadic movements (which are often undertaken as a group [30]). Thus, individuals may benefit from living in groups—and potentially offset slower movements—if the collective navigational capacity of groups allows them to follow straighter movement paths.

**Figure 1.**
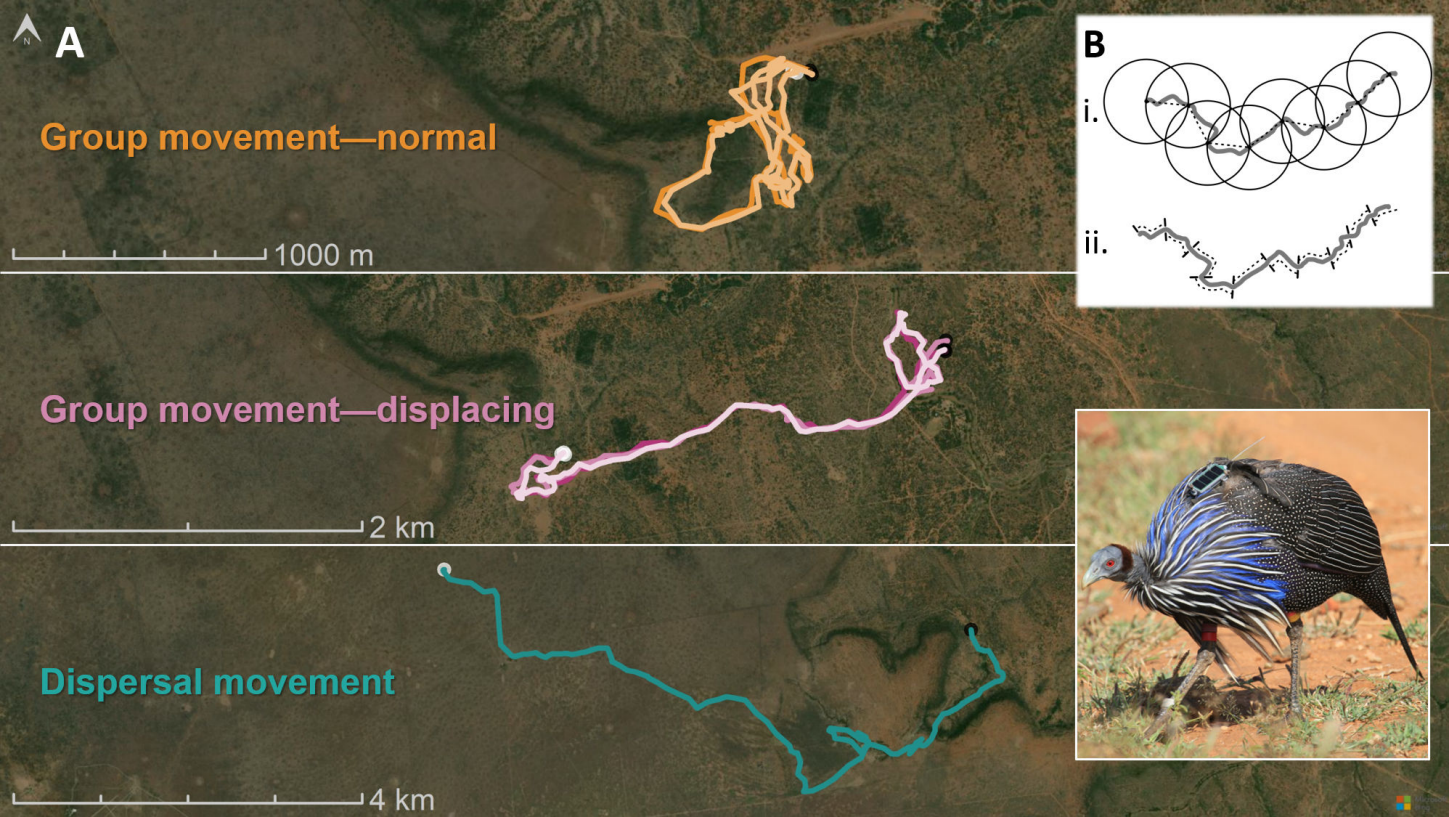
Movement of GPS-tracked vulturine guineafowl across three conditions. (A) Illustrative tracks of normal daily movements of birds moving as part of a cohesive social group (orange, top), movements made by those same groups on days when they make large displacements in search of resources (purple, middle) and large displacements made by lone individuals during natal dispersal (green, bottom). Morning and evening roosting points are marked by black and white points, respectively. (B) Illustration of how a track is segmented into 50 m units of either (i) net or (ii) cumulative displacements. The inset shows a vulturine guineafowl fitted with a solar-powered GPS tracker.

In this article, we combine high-resolution GPS tracking of vulturine guineafowl (*Acryllium vulturinum*), an almost exclusively terrestrial bird, with laboratory models of guineafowl physiology [31] to compare the cost of transport for individuals moving in groups versus alone. Specifically, we use our data to generate a two-part comparison of the movement characteristics of individuals moving as part of a collective (figure 1A). First, we compare the normal daily movements of individuals in groups to their movements on days when their group made large displacements, which are typically made during dry conditions when groups leave their preferred home ranges to search for resources [32]. This first comparison allows us to test if individuals in groups are capable of increasing the energetic efficiency of movement (i.e. having a lower cost of transport) under conditions that should favour the expression of more efficient movements. Second, we then compare these data to similar large movements of individuals moving alone through the same landscape, captured during the solitary phase of dispersal, which typically occurs at the onset of wet seasons [33]. This second comparison allows us to determine the scale of any increases in efficiency expressed by individuals moving in groups versus moving alone (i.e. when both are making large, theoretically costly displacements, testing whether individuals in groups can be as efficient as lone individuals). Across these three conditions, we also analyse the properties of individuals’ movements, thus quantifying the relative contributions of movement speed, continuity and straightness to their energetic efficiency. In doing so, we demonstrate that individuals pay greater energetic costs of transport during collective movements, arising from reductions in the speed and continuity of movement, revealing an often-overlooked cost of living in groups.

## 2. Methods

### (a) Study system

We conducted our research at the Mpala Research Centre in Laikipia, Kenya (0.292120, 36.898670), which comprises a mix of semi-arid savannah and scrubland habitats. Here, we have studied a population of vulturine guineafowl (*A. vulturinum*)—consisting of 600−1000 individuals across approximately 20 stable groups—continuously since 2016. Since the start of this study, over 1300 individuals have been identified and marked with individually numbered stainless-steel rings and a unique visual identifier—either a four-colour combination of plastic leg bands or a numbered canvas tag on the wing (for individuals captured as juveniles and too small to be fitted with rings). Birds were captured in groups, using large (8 m × 4 m × 2 m) walk-in traps to catch all members of a group at once.

#### (i) GPS tracking

A subset of individuals in each group were fitted with a 15 g Bird Solar GPS tag (e-obs GmbH), using a backpack-style Teflon harness and a foam rubber pad to elevate solar panels above body feathers (following [34]). While the exact number of tagged individuals varied during the study period, there were typically between two and five tags deployed to residents in each social group (see [35] for details on deployment strategies), with an additional 25 tags deployed on subadult females to track dispersal, resulting in approximately 100 concurrently deployed tags at any given time. In total, the approximate mass of all markings and GPS backpacks was 20.5 g, less than 2% of birds’ body mass (range: 1160−1900 g).

For the purpose of this study, we used GPS tracking data from 113 individuals, comprising 19 dispersing subadult females—approximately 18 months old at the time of dispersal—and 94 group-living residents (52 adult males and 42 adult females) distributed across all social groups in our study population. GPS data were recorded over 5 years from November 2016 to September 2021, including two periods of dispersal in April and October 2019. Importantly, because all individuals in the study (those that dispersed and those that moved in groups) were fitted with the same tags, our data are highly comparable in terms of addressing our central research question.

GPS devices were programmed to record data during daylight hours, from 06.00 to 19.00, when birds were active outside of their night-time roosts. GPS data (date, time and location) were recorded at two resolutions, depending on tags' battery level. When tags’ batteries were fully charged (approximately every second to third day), tags collected continuous 1 Hz data (i.e. one fix per second). When battery levels fell below the high-resolution charge threshold, we set tags to record a 10 s burst of 10 fixes every 5 min. The use of clustered sampling (whether continuous 1 Hz or short bursts) provides a substantial increase in fix accuracy, with higher temporal resolutions gaining further accuracy benefits as consecutive fixes that are closer in either space or time are generally more accurate. For example, the error for the 10th fix in a burst is <4 m, and with error also correlated in time, the relative movement caused by error in consecutive fixes is minimal (<1 m; see [35] for further details of tag accuracy under field conditions). If battery charge was at the lowest threshold, tags were set to record one point every 15 min (this threshold was not crossed during this study).

Data were remotely downloaded every 2 days (sometimes daily in the case of actively dispersing birds) using a BaseStation II (e-obs Digital Telemetry, Grünwald, Germany). Data were separated into two resolutions for analytical purposes: high-resolution data, comprising all continuous periods of 1 Hz data; and 5 min data, comprising data from the tenth second of every fifth minute of the day. The latter was collected from both the low-resolution dataset and by subsampling the 1 Hz data, thereby reliably providing one fix every 5 min for every bird on every day of tracking. Accelerometer functions of GPS tags were disabled for this study, as the increased amount of data would have hindered our ability to reliably download data at regular intervals, given the number of tags concurrently deployed. GPS data were uploaded to Movebank (https://www. movebank.org/) and retrieved and prepared for analysis in R using the move package [36].

### (b) Analysis

All analyses were performed in R v. 4.0 [37].

#### (i) Identifying large-scale movements

In order to draw comparisons between movements of grouped individuals and lone dispersers, we isolated all days within our dataset of GPS movements that comprised a large daily displacement. On most days, groups of guineafowl return to the same roost, or a nearby roost, resulting in small daily displacements. However, at the onset or end of extreme weather conditions (e.g. droughts), groups move out of (or back into) their regular home range [32]. During such days, groups make large displacements, which are qualitatively similar to those made by actively dispersing individuals in terms of covering large distances over the course of a day, resulting in similarly large roost-to-roost distances. These movements are also made through similar habitats as both groups and lone dispersers navigate their way outside of the same core study area. Following [33], we identified days that primarily contained large-scale movements based on the length and straightness of each individual’s daily movement path—if either the roost-to-roost distance (i.e. daily net displacement) was greater than 1500 m, or if the roost distance was greater than 1200 m and the ratio of the distance between roosts and their total daily track length was greater than 0.3. The latter captures days of large movements, where groups or individuals made a large turn, thereby reducing the roost-to-roost distance but maintaining a large overall movement distance. These same criteria were used for isolating the solitary movements of dispersers, as transience in this species typically entails two modes: active dispersal, where individuals make large solitary movements, and more local days of movement, which observations suggest correspond to periods of movement within groups other than their natal or post-settlement group [33].

For grouped individuals, we also included a matched set of ‘normal’ days of movement to test for changes in the cost of transport (and subsequent efficiency benefits) on days of extreme movements. For each individual, we selected *N* days at random from the remaining dataset, where *N* was the number of days of large movements recorded from that individual. For lone dispersers, we only used days of extreme movement to facilitate comparison with grouped birds, as overall changes in movement during dispersal were characterized in [20].

#### (ii) Defining movement states

We employed an unsupervised Hidden Markov Model (HMM) to identify the different states of movement exhibited by vulturine guineafowl. Movement states were characterized by first summing the distance moved and absolute turning angles for every 10 s in the high-resolution data—i.e. maximizing temporal resolution to avoid capturing multiple behaviours in a single window while avoiding violations of the Markov assumption (which would be likely if using 1 Hz data directly). Based on our previous work [20], we separated movement into four states across the entire high-resolution dataset, using the R package depmixS4 [38]. The choice to use a four-state model was based on field observations that individuals spend time not moving (state 1), making slow, tortuous foraging movements (state 2), walking at a medium speed (state 3) and moving quickly in a directed manner (state 4). A critical reason for the implementation of the four-state model was the need to isolate a clear ‘stationary’ state—which was not clearly recoverable with a three-state model—for the purposes of calculating metabolic expenditure, with birds considered to be moving when assigned to states 2−4. We fitted the HMM models with three categorical labels (grouped individuals making extreme movements, grouped individuals moving normally and solitary dispersers) as a covariate on the state transition matrix (see electronic supplementary material, table S1 for state distributions and transition probabilities). The large-scale movements of grouped individuals were treated as the reference category when fitting state transition probabilities. Using the predictions of the HMM, we then assigned states to each 10 s cluster of data and, eventually, to every second in the 1 Hz data that comprised each cluster.

#### (iii) Characterizing movement behaviours

We extracted four representative measures to characterize birds’ movement behaviours: daily track length (km), speed while moving (m s^−1^) and the straightness of movement (a straightness index, [39]) at two temporal resolutions—daily and at finer 5 min intervals (see electronic supplementary material, table S2 for additional temporal resolutions). The two temporal measures of straightness capture how directed their movements were over the whole day versus how directed they were as they navigated through local habitat. To account for variation in high-resolution data collected across different days, daily track lengths were measured from the sum of displacements in the 5 min data (which was available for every bird on every day). Movement speeds were calculated as the speed-while-moving (i.e. from all data assigned to states 2−4) based on the per-second displacement and then summarized for each 5 min window of available high-resolution data (restricted to windows with >50% high-resolution coverage). To reduce the effect of small GPS errors on calculated velocities, we derived individual speeds at each second from the mean velocity over a rolling 5 s window within the high-resolution data (i.e. for each second of 1 Hz data, we averaged the individual’s speed with the 2 s that preceded and followed it). Straightness of movement was characterized by dividing the net displacement achieved over a given time interval by the summed cumulative displacement therein. For daily intervals, this was achieved by dividing the distance between the first and last point of each individual’s daily data by the sum of displacements in the 5 min data. For fine-scale intervals, we calculated the net displacement achieved in each available 5 min window (or other time windows; electronic supplementary material, table S2) of 1 Hz data by the summed second-by-second displacements within that time window (restricted to windows with >95% high-resolution coverage).

To test how the behaviours of individuals in groups varied during periods of extreme movement, we fit linear mixed models (LMMs) (using the package lmerTest [40]) to our measures of (log-transformed) track lengths and speeds, and fit beta regressions (using the package glmmTMB [41]) to our measures of straightness at both daily and finer time scales. When analysing daily track length and day-level straightness, we considered one measure per individual per day. Because the distribution of step lengths is left-truncated, forming a long-tailed distribution, we log-transformed movement speed and daily track length values to aid with model fitting. For fine-scale analyses of movement speed and straightness, we included one observation—i.e. log-transformed mean speed-while-moving within a given window, and the straightness index over the full window—from each available 5 min window of continuous high-resolution data (mean = 69.4, range = 4 to 156 observations per individual per day). In each model, we included the context (i.e. group member during a large displacement, group member on a normal day or lone disperser) as a predictor and individual identity as a random intercept. For models of movement speed and fine-scale (5 min) straightness of movement, we also included the time of day (binned hourly) as an additional random intercept. We additionally conducted a sensitivity analysis around the temporal binning by fitting models based on the speed and straightness of movement summarized at several temporal resolutions—ranging from 30 s to 1 h (electronic supplementary material, table S2). All regression models were fit using maximum likelihood estimation. The specific structure for all models can be found in the corresponding table in the supporting information (electronic supplementary material, tables S3–S6).

#### (iv) Calculating the energetic cost of movement

To quantify the energetic costs of movement, we used published data on the relationship between metabolic costs (ml O_2_ kg^−1^ s^−1^) and movement speed (m s^−1^) in the closely related, and morphologically similar, helmeted guineafowl (*Numida meleagris*) moving at set speeds and inclines in a controlled laboratory setting [31]. We calculated the costs incurred for each second of high-resolution data according to whether birds were either stationary (state 1) or moving (states 2−4). When moving, guineafowl exhibit a linear relationship between speed (i.e. velocity v > 0) and oxygen consumption, the slope and intercept of which vary depending on the incline at which birds are moving [31]. This relationship takes the form of ${VO}_{2}=24.0v+27.2$ on level terrain, ${VO}_{2}=30.7v+27.6$ at 10% incline and ${VO}_{2}=47.7v+21.3$ at 20% incline [31], where VO_2_ is the per-minute volume of oxygen consumed in ml O_2_ per kilogram of body mass (ml O_2_ kg^−1^ min^−1^). While some energetic savings can be achieved from downhill movements, these are often offset by increased muscle usage to absorb greater impact [42]. Thus, downhill movements provide either minimal savings, especially at slower speeds (e.g. in humans [43]) or may even slightly increase the cost of transport (e.g. in barnacle geese *Branta leucopsis* [42]) relative to level ground. As there were no specific studies on guineafowl, we assumed all downhill movements followed the same cost relationship as movements on level ground.

In order to determine the incline of the terrain that birds were moving along, we obtained a digital elevation model (DEM) from the Open Data Site of the Regional Centre for Mapping of Resource for Development (opendata.rcmrd.org) with a spatial resolution of 30 m (elevation values were ground-truthed by taking measures at 24 locations within our study area). The slope and aspect (i.e. the compass direction of the vector representing the greatest slope) of the terrain in each cell of the DEM were calculated in degrees based on the surrounding eight neighbouring cells using the ‘terra’ package [44], following [45]. We then calculated the degree of the slope experienced by each bird in each step in our 1 Hz data based on the relationship between the individual’s movement bearing and the aspect of the incline following the formula $\theta^{\prime }=\tan^{-1}\left(\tan\left(\theta \right)\;\cdot \;\cos\left(\Delta \psi \right)\right)$, where $\theta^{\prime }$ is the slope experienced by the individual, θ is the slope of the inclined plane and ∆ψ is the angular difference between the aspect of the incline and the individual’s bearing. Experienced slope values were transformed into per cent grade by the formula $PG=\tan\left(\theta^{\prime }\right)*100$ where PG is the per cent grade of an incline corresponding to the slope experienced by an individual (in degrees). For the purposes of aligning incline values with formulas for oxygen consumption, the per cent grade at each GPS fix was rounded to the nearest 10 and birds were considered as moving on level terrain when the resultant value was less than 5% (i.e. rounded to 0), were considered as moving at 10% incline when the rounded value was between 5 and 15%, and followed the formula for a 20% incline when resulting values were ≥15% (note that <0.001% of data correspond to an experienced grade of 30% or more). The formula for when birds were stationary is a fixed consumption of 19.1 ml O_2_ kg^−1^ min^−1^, corresponding to the oxygen consumption rate when not moving (i.e. the resting metabolic rate), as described in the literature [31]. We then transformed the per-second measures of metabolic oxygen consumption into units of Joules kg^−1^ s^−1^ using a conversion factor of 20.1 J ml^−1^ O_2_ [31,46].

#### (v) Calculating the energetic cost of transport

To relate energetic expenditure to achieved displacements (i.e. the cost of transport), we partitioned movement tracks in our high-resolution data into fixed segments representing 50 m of net or cumulative displacement (figure 1B). Net displacement is the absolute movement in space (i.e. a straight-line distance) between two points in time, while cumulative displacement is the summed travel distance of all individual steps. The cost of transport as measured relative to a given net displacement captures the contributions of both movement speed and path straightness to efficiency, while the cumulative displacement measure captures the effect of speed irrespective of straightness. We defined net displacement segments starting from the first available second of high-resolution data (i.e. after the GPS switched on or switched from low-resolution to high-resolution) until the 50 m net displacement threshold was crossed. We then used the first GPS point to fall on or outside of the 50 m radius as the first point for the following segment. We also calculated the corresponding cumulative displacements from the same high-resolution data by summing each consecutive step length in a track until it reached a sum of 50 m, at which point we started a new segment. We then summed the per-second costs for each detection that contributed to a segment to calculate the total energetic expenditure for that segment, and translated it into the energetic cost of transport (in J kg^−1^ m^−1^) for each type of displacement by dividing by the distance travelled (either net or cumulative). Because segments varied slightly from perfect, 50 m denominations, cost of transport values were calculated relative to the true distances travelled.

For each displacement type, we fit separate LMMs with the cost of transport associated with each segment as the response variable, the context as a predictor, and individual identity and time of day (binned hourly) as a random intercept. Model formulations can be found in corresponding supplementary tables (electronic supplementary material, tables S7 and S8).

#### (vi) Calculating cumulative daily energy use

We calculated the total energetic expenditure over each 13 h day for each bird by summing all of the per-second costs from each unique day of tracking for each individual into a single measure of daily energetic expenditure (i.e. J kg^−1^ d^−1^). Not all days contained an equal amount of high-resolution data (average of 5.75 h of high-resolution data on days when high-resolution data were recorded), and thus we standardized the cost value for each day by multiplying the mean per-second cost within the day by 46 800 s. Days containing fewer than 2 h of high-resolution data were excluded from this analysis, to avoid potentially over-representing days with too little high-resolution data.

To estimate the change in energy use across contexts, we fit an LMM of daily energetic expenditure as the response variable, with context as the predictor variable and individual identity as a random intercept. The model formulation can be found in electronic supplementary material, table S9.

## 3. Results

### (a) Individuals moving in groups increase their energetic efficiency when making large displacements

**Figure 2.**
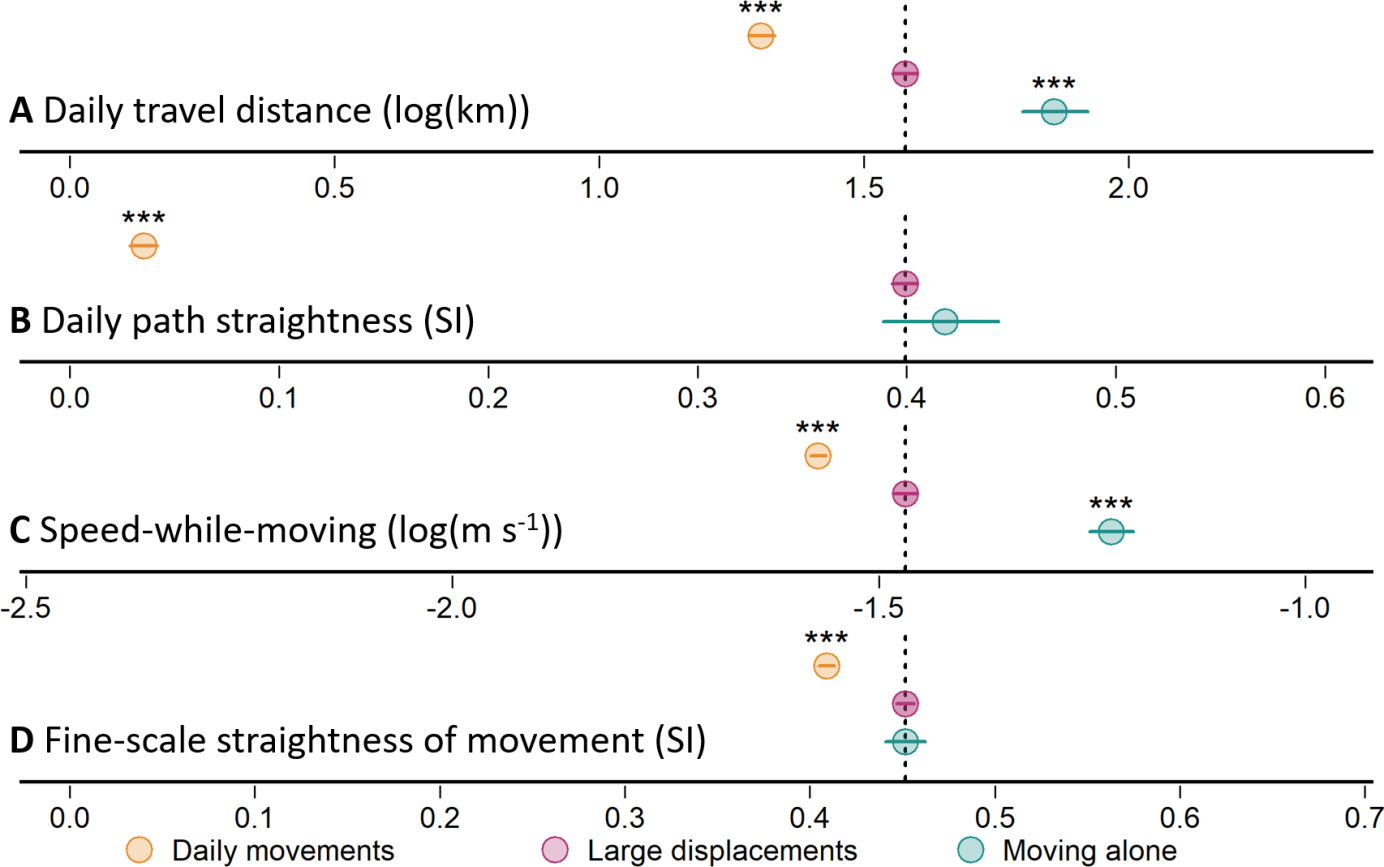
Birds moving in groups moved farther, faster and straighter when making large displacements, but lone individuals moved even further and faster. (A–D) Summaries of regression models characterizing (coefficient ± 95% confidence intervals) movement behaviours when individuals made daily movements with their group, made large displacements in a group and moved alone during dispersal. Significance (***p < 0.001) was estimated using large displacements of individuals in groups as the reference category (dotted vertical lines correspond to model intercept). Speed-while-moving (C) and fine-scale straightness (D) are calculated over 5 min windows (additional temporal resolutions in electronic supplementary material, table S2). Full model results are available in electronic supplementary material, tables S3–S6.

**Figure 3.**
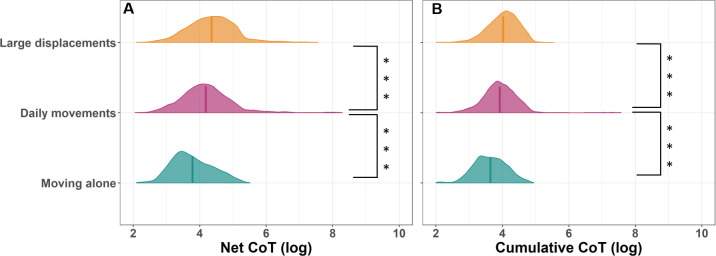
The cost of transport (CoT) is significantly reduced when individuals make large displacements relative to regular daily movements, but individuals moving in groups are less efficient than those moving alone. Plots show log-transformed distributions of expressed costs of transport for individuals making (A) a 50 m net displacement and (B) a corresponding cumulative displacement to achieve 50 m of net displacement. Vertical lines show the mean cost of transport values per category and statistical significance in differences between categories marked with asterisks (***p < 0.001; see electronic supplementary material, tables S7 and S8 for full model results).

We extracted 886 days of large daily displacements from population-scale GPS tracking starting in September 2016 until September 2021. When moving as part of a group, individuals moving in groups expressed an average 23.9% increase in daily travel distances (*p* < 0.001, figure 2A; electronic supplementary material, table S3) and a 91.1% increase in daily path straightness (*p* < 0.001, figure 2B; electronic supplementary material, table S4) on days of large movements relative to their normal movements. On these days, individuals were also more energetically efficient, expressing an average 15.3% reduction in the net cost of transport (*p* < 0.001) and a 7.4% reduction in the cumulative cost of transport (*p* < 0.001) relative to days of normal daily movements (figure 3; electronic supplementary material, tables S7 and S8). As a result of moving with greater efficiency, large displacements resulted in only a 2.9% increase in total daily energy expenditure (*p* < 0.001; electronic supplementary material, figure S1, table S9).

### (b) Individuals moving in a group are less efficient than individuals moving alone

Comparing 96 days of large dispersal movements by individuals moving alone with the large movements of individuals moving in groups shows that individuals moving in groups were significantly less efficient than lone dispersers. When moving alone, dispersers travelled on average 32.5% further per day than individuals making large displacements in groups (*p* < 0.001, figure 2A; electronic supplementary material, table S3) and exhibit similar daily path straightness (4.7% increase, *p* = 0.195, figure 2B; electronic supplementary material, table S4). However, individuals dispersing alone had a significantly lower net cost of transport (35.7%, *p* < 0.001, figure 3A; electronic supplementary material, table S7) and cumulative cost of transport (25.9%, *p* < 0.001, figure 3B; electronic supplementary material, table S8) than individuals making large displacements in a group.

### (c) Individuals moving in groups make slower, more sporadic movements

An analysis of fine-scale movement behaviours revealed that individuals moving in groups expressed distinct differences in behaviour, relative to individuals moving alone when making large displacements. The increased energetic efficiency of group members’ large movements was the result of a significant increase in (5 min) average speed-while-moving (9.7%, *p* < 0.001, figure 2C; electronic supplementary material, table S5), and a significant increase in the average fine-scale (5 min) straightness of movement (9.4%, *p* < 0.001, figure 2D; electronic supplementary material, table S6). This pattern also bore out in our sensitivity analysis, where group members made consistently faster (range: 9.0–9.9% increase; electronic supplementary material, table S2) and straighter (range: 2.9–20.3% increase; electronic supplementary material, table S2) movements when engaging in large displacements, including when straightness was measured at different time scales. However, lone individuals were able to maintain greater movement speeds than individuals moving in a group (27.2% greater, *p* < 0.001, figure 2C; electronic supplementary material, table S5), while there was no clear difference in the (5 min) fine-scale straightness of movement (0.002% lower than group members, *p* = 0.99, figure 2D; electronic supplementary material, table S6). In our sensitivity analysis, lone dispersers were consistently faster than group members when making large displacements (range: 26.8–29.4% increase; electronic supplementary material, table S2). However, the difference in fine-scale straightness of movement varied with temporal resolution, with lone dispersers exhibiting straighter movements than group members at the finest (30–60 s) time scales, no significant difference at intermediate (2–10 min) time scales and group members exhibiting straighter movements at the coarsest (15–60 min) time scales (electronic supplementary material, table S2). However, when making large displacements, both lone dispersers and group members exhibited consistently straighter movements than those exhibited by group members during normal daily movements across all time scales.

Categorical differences in state transition probabilities within our HMM revealed that individuals moving in groups also had a substantially lower likelihood of maintaining the most efficient movement state (65.2% probability of remaining in state 4, which corresponds to the fastest, straightest movements; electronic supplementary material, table S1) than individuals moving alone (75.2% probability of remaining in state 4; electronic supplementary material, table S1). In general, group members showed a greater probability of transitioning from ‘slower’ to ‘faster’ states (e.g. transitioning out of the stationary first state or transitioning to states 3 and 4 from state 2) during days of large movement than they did during normal daily movements (electronic supplementary material, table S1), but this general probability of transitioning to higher states was greater for lone dispersers.

## 4. Discussion

Our study shows that group living can have a significant, constraining effect on the energetic efficiency of movement for individuals that move as part of a group. While individuals are on average more efficient when making large displacements in a group relative to their typical daily ranging movements in the same groups, they do not move as fast or as continuously as individuals that make similarly large displacements alone. This suggests that individuals are better able to express more efficient movement strategies—specifically reducing the cost of transport by moving faster—when making large displacements alone than when doing so as part of a group. These results reveal that group living is likely to generate greater mobility costs for individuals.

While many studies on collective movement have focused on the differences in behaviour expressed by groups of different sizes [47,48], few have compared individuals in groups versus solitary individuals. In part, this is due to the inherent challenges in trying to study group-living animals making ecologically relevant movements without their group. Here, we were able to leverage a dataset spanning multiple life-history stages to draw direct comparisons between the behaviours of individuals moving in groups versus dispersers that moved alone. While these movements represented distinct life stages (being in a group versus dispersing), the two sets of movements do overlap. Vulturine guineafowl exhibit delayed dispersal (in our dataset, between 1.5 and 2.5 years post-hatching), meaning that their movements are made after they have gained substantial life experience and generally reached adult body size (females reach 90% of their adult body size at 1 year of age, DR Farine, 2024, unpublished data). Further, groups will make large displacements even when they contain females that have not yet dispersed, despite limitations to their daily displacements when groups have young chicks [47]. Thus, we expect that the observed differences do not reflect changes associated with physiology or development *per se*.

Our results raise the question: what aspects of movement behaviour can individuals optimize when alone and which are limited when in a group? A previous study on the same species [20] found that dispersing individuals increase the speed and straightness of their movements by 27.7 and 10.3%, respectively, when compared with their normal non-dispersing movements. Studies in other systems [17–19] reported similar increases in both movement speed and straightness (and greater increases in speed than straightness)—such as a 23.9% increase in speed compared with a 10.3% increase in straightness in dispersing lions (*Panthera leo*) [16]. In vulturine guineafowl, individuals moving in groups exhibited comparable increases in path straightness to individuals moving alone (at both fine-scale and larger daily time scales), but achieved a substantially lower increase in speed (9.7%) relative to their regular daily movements. These results suggest that speed is the main limiting factor for the efficiency of individuals in groups, more so than the straightness of their paths. Previous work on baboons has demonstrated how preferred movement speed within a group is in conflict among group members [10], with individuals either having to move faster when trailing behind or else stopping-and-starting if they outpaced the group. These patterns have consequences for energy efficiency. While slower individuals increase their speed (and thus overall efficiency), mechanical (e.g. limb length) disadvantages mean that they are likely to experience diminishing returns in energetic efficiency as they move beyond an efficient gait. At the other end of the spectrum, faster individuals have to repeatedly pause their movements, introducing efficiency costs [49,50].

While we demonstrated substantial losses in efficiency by individuals moving in groups as a result of lower movement speeds than individuals moving alone, the differences may have been even greater if we were able to also estimate the costs resulting from reduced continuity of movement. The state transitions in our HMM suggested that individuals in groups were less continuous in their movements, stopping and starting more frequently than solitary individuals (captured by a lower probability of remaining in the fastest movement state and higher probability of transitioning to the stationary state; electronic supplementary material, table S1). Continuity of movement is likely to be affected by the process of collective decision-making (i.e. pausing to wait for others). Groups face significant coordination challenges, and most groups resolve conflicts over where to move by making so-called shared decisions (i.e. voting for a direction) [9,11,51,52]. Previous work using GPS to track the movement of baboon troops found that individuals are less likely to move when, for example, there is a larger conflict over the proposed directions of travel [9,53]. The consequence of having to slow down and, potentially, stop to make these decisions should exacerbate the loss of efficiency that we detected. Recent work in humans, for example, found that making shorter bouts of movements by humans could result in a 20–60% increase in energetic expenditure relative to moving at the same speed in a continuous way [50]. Our results, therefore, highlight some potential hidden costs of collective movements.

Studies comparing collective navigation between lone individuals and groups (e.g. in homing pigeons) have suggested that groups should experience navigational benefits that consistently reduce their overall travel distances [6,27,28,54]. These benefits are predicted to arise from the many-wrongs hypothesis, where the error in individuals’ directional estimates cancels out to produce a more accurate average when integrated at the group level [7,26]. We found that individuals did not exhibit straighter overall (daily) paths when making large displacements as part of a group relative to moving alone. This finding raises the question of whether vulturine guineafowl do not benefit from collective navigation. Our sensitivity analysis did reveal some differences in the straightness of movement between group members and lone dispersers at different fine-scale temporal resolutions (when making large displacements). Specifically, group members made straighter movements over longer time scales (15–60 min) and lone dispersers exhibited the straightest movements at very short scales (30–60 s). This pattern could suggest some effects of directional conflicts affecting the ability of group members to express straightness at short time scales but group benefits being expressed when collectively navigating through patches of local habitat over several minutes. However, our ability to draw strong conclusions on lone dispersers versus group members making large displacements is limited because we had substantially fewer complete windows of high-resolution data from lone dispersers over longer time scales (i.e. > 15 min with 95% coverage). Importantly, when making large displacements, both group members and lone dispersers consistently exceeded the straightness of group members making typical daily movements, and the differences in straightness between group members and lone dispersers do not play out at a daily scale.

Several factors could shape the straightness of the paths taken by animals during large displacements. Goal-oriented movements are generally assumed to result in straighter paths towards a known destination [7,55]. However, dispersing individuals are generally searching for settlement sites in new areas. Straightness could instead emerge during dispersal because this is one of the most effective strategies for sampling habitats [56] (although this utility can break down under energetic constraints [56], and does not account for the notion that naive individuals may be more likely to encounter movement barriers [57]). Some dispersers also failed to settle into a new group and returned back to their natal group (four recorded cases), and these movements are likely to be more goal oriented. Ecological context is also important when groups make large displacements, which is typically in response to harsher environmental conditions driving them to depart from their preferred home ranges [32]. Thus, outward movements by large group movements may also entail some level of exploration (i.e. searching for areas with better resources), with return movements (back to the preferred home range) being more goal oriented. Recent work suggests that groups move significantly faster and straighter when returning to their preferred home range (typically after rainfall events) than when they make more exploratory movements [58]. Thus, both lone dispersers and group members making large displacements are likely to express a mix of exploratory and goal-oriented movements. More systematic investigations during periods of goal-directed versus exploratory movements may generate new insights into the differing factors—possibly beyond just energetic efficiency—driving the expression of similar behavioural strategies across different life stages.

A further factor that could modulate movement behaviours during large displacements is the differing habitat selection pressures acting on dispersers and residents. This again is a multidimensional topic. For example, several studies have shown that animals tend to select different habitats during large movements than those they typically reside in or forage in [59,60]. However, if animals are searching for foraging resources (as was likely the case for the large group movements in our study), then they may need to move through more resistant habitats to do so. By contrast, dispersing guineafowl must locate other groups to enter. While this might also force them into residential habitats, the range at which a group can be detected (e.g. via auditory cues like contact calls) is possibly large enough to allow dispersers to focus on moving through the most suitable habitats without sacrificing sampling opportunities. Recent work on vulturine guineafowl has suggested that dispersers disproportionately select more open habitats and roads because these provide substantial benefits in terms of movement speed and efficiency [61]. These same benefits should also be sought for by guineafowl groups when making large movements. The funnelling effect of habitat [62] may, therefore, shape both types of movements in similar ways, thereby leading to similar movement paths.

Despite the clear costs that individuals pay in terms of their energetic efficiency when moving as a group, group living could still bring substantial benefits in other ways. One major energetic cost for many vertebrates is brain power [63–65], and moving as a group could modify these costs. Examples of lone ants and pigeons being unable to recapitulate routes after following others [66,67] suggest that followers may be able to shift their cognitive resources from landscape features to focus on other factors, such as the behaviour of conspecifics or watching for predators. Given that cognitive load can be measured as birds navigate through landscapes [68], future studies could compare the cognitive costs paid by individuals when navigating large displacements alone versus in a group. Similarly, biologging advances in heart rate monitoring [69,70] could also potentially reveal whether individuals pay lower physiological costs (e.g. maintaining a lower heart rate) when moving in a group versus alone. There are several ways this could happen, one being because they are less sensitive to risk [71,72], and another because following can reduce the heart rate when moving (drafting during races is a classic example in humans [73,74]). These complex interactions of risk [75,76], energetics [77,78], resources [2,79], and social structures [80] all provide fertile ground for future research into how group living modifies the fundamental ecology of movement in social species.

## Data Availability

All data and code necessary to replicate this study can be found at [81]. Supplementary material is available online [82].
