# Correction to: ‘Moving as a group imposes constraints on the energetic efficiency of movement’ (2025) by Klarevas-Irby *et al*

**DOI:** 10.1098/rspb.2025.0692

**Published:** 2025-04-09

**Authors:** James A. Klarevas-Irby, Brendah Nyaguthii, Damien Farine

**Affiliations:** ^1^ Department of Migration, Max Planck Institute of Animal Behavior, Radolfzell, Germany; ^2^ Department of Evolutionary Biology and Environmental Studies, University of Zurich, Zürich, Switzerland; ^3^ Division of Ecology and Evolution, Research School of Biology, Australian National University, Canberra, Australia; ^4^ Mpala Research Center, Nanyuki, Kenya; ^5^ Department of Ornithology, National Museums of Kenya, Nairobi, Kenya; ^6^ Department of Collective Behavior, Max Planck Institute of Animal Behavior, Konstanz, Germany

*Proc. R. Soc. B*. 292, 204110. (Published online 19 February 2025) (https://doi.org/10.1098/rspb.2024.2760).

In figure 3 of the original published version [1], the *y*-axis labels for ‘Large displacements’ and ‘Daily movements’ were incorrectly swapped with one another. The plotted data and figure legend were correct, as the error stemmed from a mis-ordering of variables in the single line of code which generated the axis labels. The correct, updated figure should be as shown.

**Figure 3.**
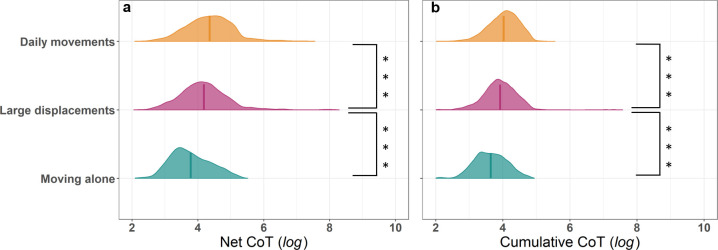
The cost of transport (CoT) is significantly reduced when individuals make large displacements relative to regular daily movements, but individuals moving in groups are less efficient than those moving alone. Plots show log-transformed distributions of expressed costs of transport for individuals making (*a*) a 50 m net displacement and (*b*) a corresponding cumulative displacement to achieve 50 m of net displacement. Vertical lines show the mean cost of transport values per category and statistical significance in differences between categories marked with asterisks (***p < 0.001; see electronic supplementary material, tables S7 and S8 for full model results).
